# Supplementary material for: Microbiota alters the metabolome in an age- and sex- dependent manner in mice
Source: Nat Commun. 2023 Mar 11;14:1348. doi: 10.1038/s41467-023-37055-1 (PMC10008592; doi:10.1038/s41467-023-37055-1)
Supplement: Supplementary file 1 — Supplementary Information [file 41467_2023_37055_MOESM1_ESM.pdf]

Microbiota alters the metabolome in an age- and sex- dependent manner in mice

Kirsty Brown[1], Carolyn A. Thomson[1], Soren Wacker[2], Marija Drikic[2], Ryan Groves[2], Vina Fan[1], Ian A. Lewis[2], Kathy D. McCoy[1*]

[1]Dept. of Physiology and Pharmacology, Snyder Institute of Chronic Diseases, Cumming School of Medicine, University of Calgary, Calgary, T2N 4N1, Canada.

[2]Dept. of Biological Sciences, University of Calgary, Calgary, T2N 1N4, Canada

*Correspondence: kathy.mccoy@ucalgary.ca

This supplemental data file contains:

Supplemental Figures 1-8

Supplemental Table 1-2

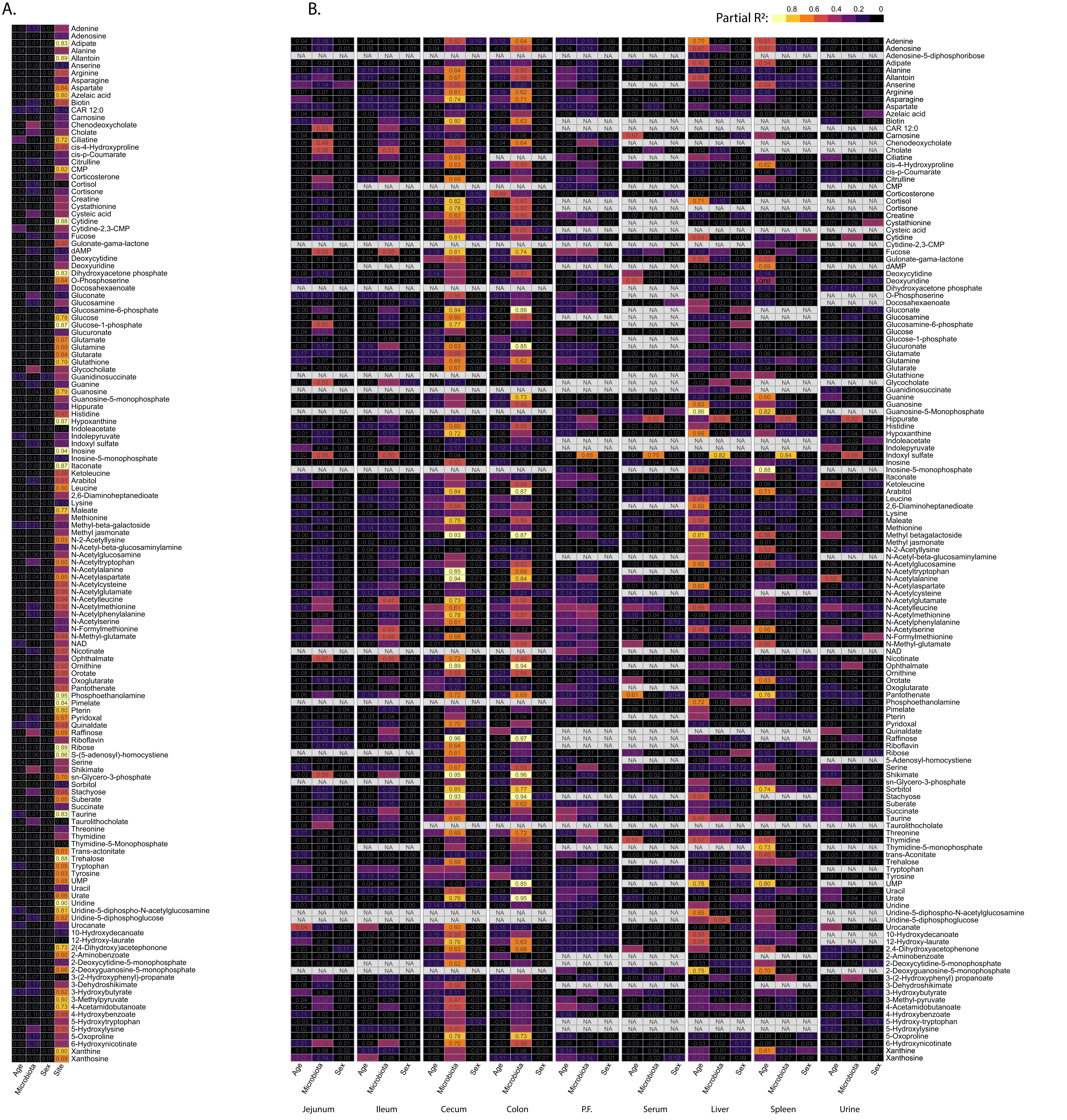

Supplementary Figure 1. Partial effect size (partial R2) of each variable attributable to factors. (A) All sites together showing the effect size attributable to age, microbiota, sex and site for each metabolite. (B) Analysis of individual sites showing the effect size attributable to age, microbiota and sex for each metabolite. NA = not applicable (metabolite detected in <80% of samples from the site); P.F = peritoneal fluid. Data is representative of n=72 samples / site with equal representation from male and female mice, GF, OMM12 and SPF colonized mice and 3-, 8- and 12-week-old mice. Source data are provided as a source data file.

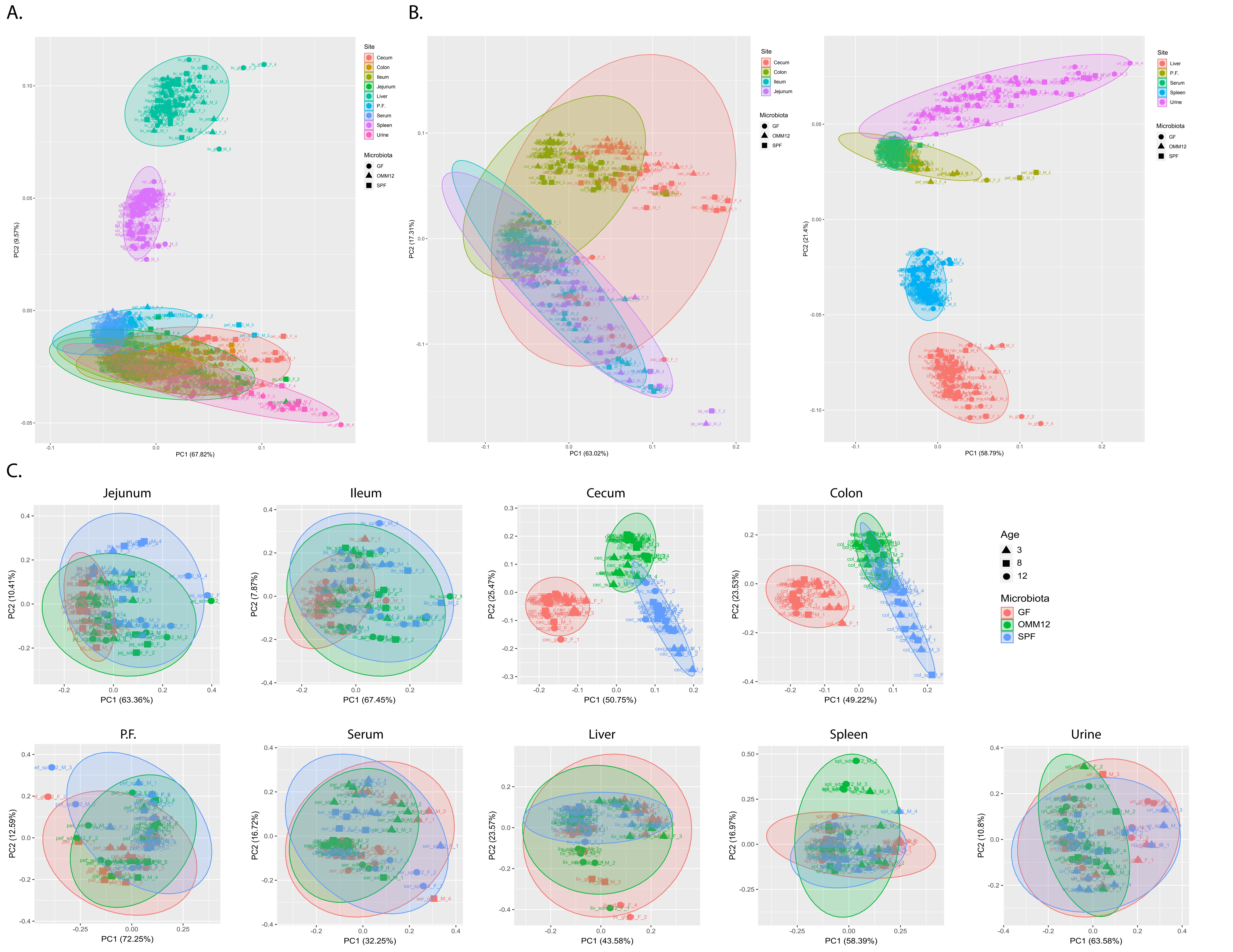

Supplementary Figure 2. Metabolite profiles shown by principal component analysis. (A) Metabolite profiles in all sites (A), (B) within the intestine and at systemic sites (B) and (C) at each individual site (C). Data were normalized using z-scores and Euclidean distance was used to determine distance. Data is representative of n=72 samples / site with (equal representation from male and female mice, GF, OMM12 and SPF colonized mice and 3-, 8- and 12-week-old mice). GF=germ-free, OMM12=Oligo-MM12, SPF=specific pathogen free, P.F. = peritoneal fluid. Source data are provided as a source data file.

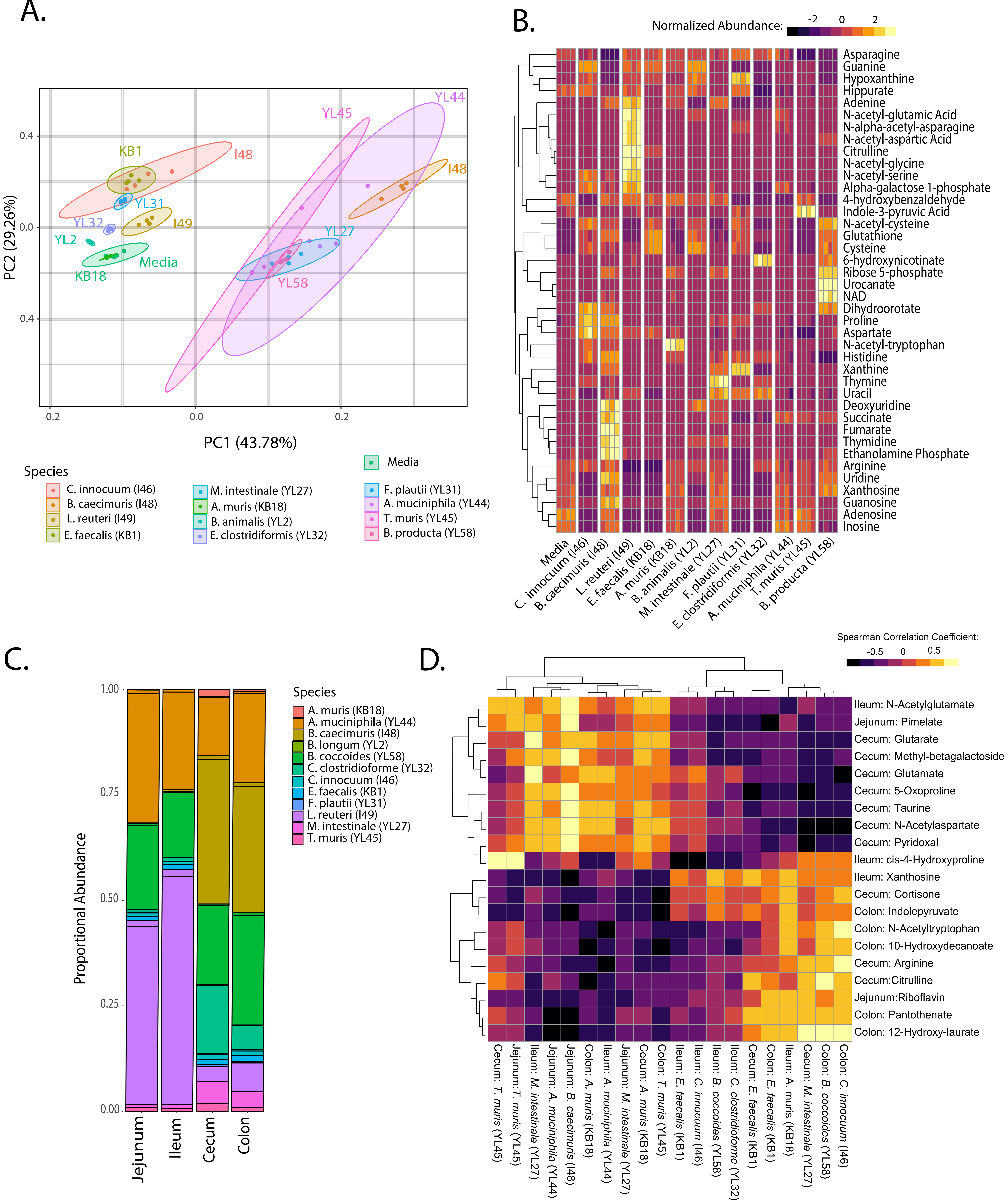

Supplementary Figure 3. OMM12 metabolite profiles of commensal bacteria in vitro and microbe-metabolite connections in vivo. (A) Metabolite profiles from culture supernatants of the members of the OMM12 consortia and (B) heatmap showing the 40 metabolites that were most significantly altered based on species. Data for each metabolite were normalized (z-score) across all samples. (C) Bacterial community profiles in the jejunum, ileum, cecum and colon of OMM12 colonized mice. (D) Twenty metabolite abundance and twenty microbe abundance variables within the GIT of adult OMM12 colonized mice that have the strongest spearman correlation values. Bacterial abundance was centre log ratio transformed and metabolites abundance was z-score normalized prior to spearman correlation coefficient calculations. Bacterial culture samples represent n=4 individual cultures per species. In vivo data is representative of n=16 mice with equal representation from 8- & 12-week-old mice and male and female mice. Source data are provided as a source data file.

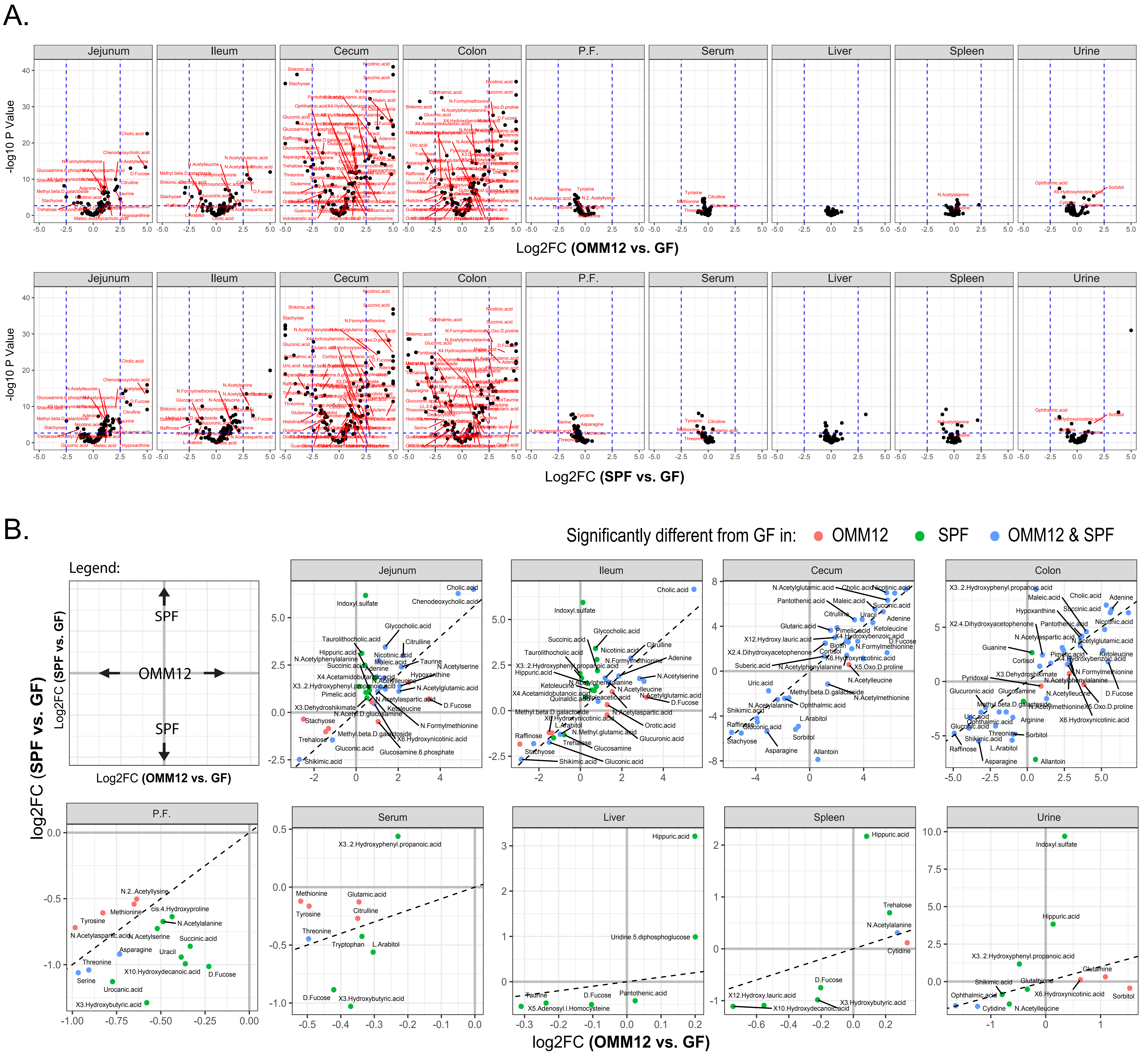

Supplementary Figure 4. Impact of microbial composition on metabolism (related to Figure 3). (A) Volcano plot showing differentially abundant metabolites in OMM12 vs. GF and SPF vs. GF. Labelled metabolites are significantly different (PAdj < 0.05) or Abs(Log2FC > 2.5). (B) Labelled biplot showing the Log2FC in SPF vs. GF and OMM12 vs. GF in metabolites that were significantly different (PAdj < 0.05) from GF in either colonization (red dots = OMM12, green dots = SPF) or both colonizations (blue dots). Data represents 24 mice per group with equal representation from male and female mice and mice that are 3-, 8-, and 12- weeks of age. GF=germ-free, OMM12=Oligo-MM12, SPF=specific pathogen free, P.F.=peritoneal fluid. Source data are provided as a source data file.

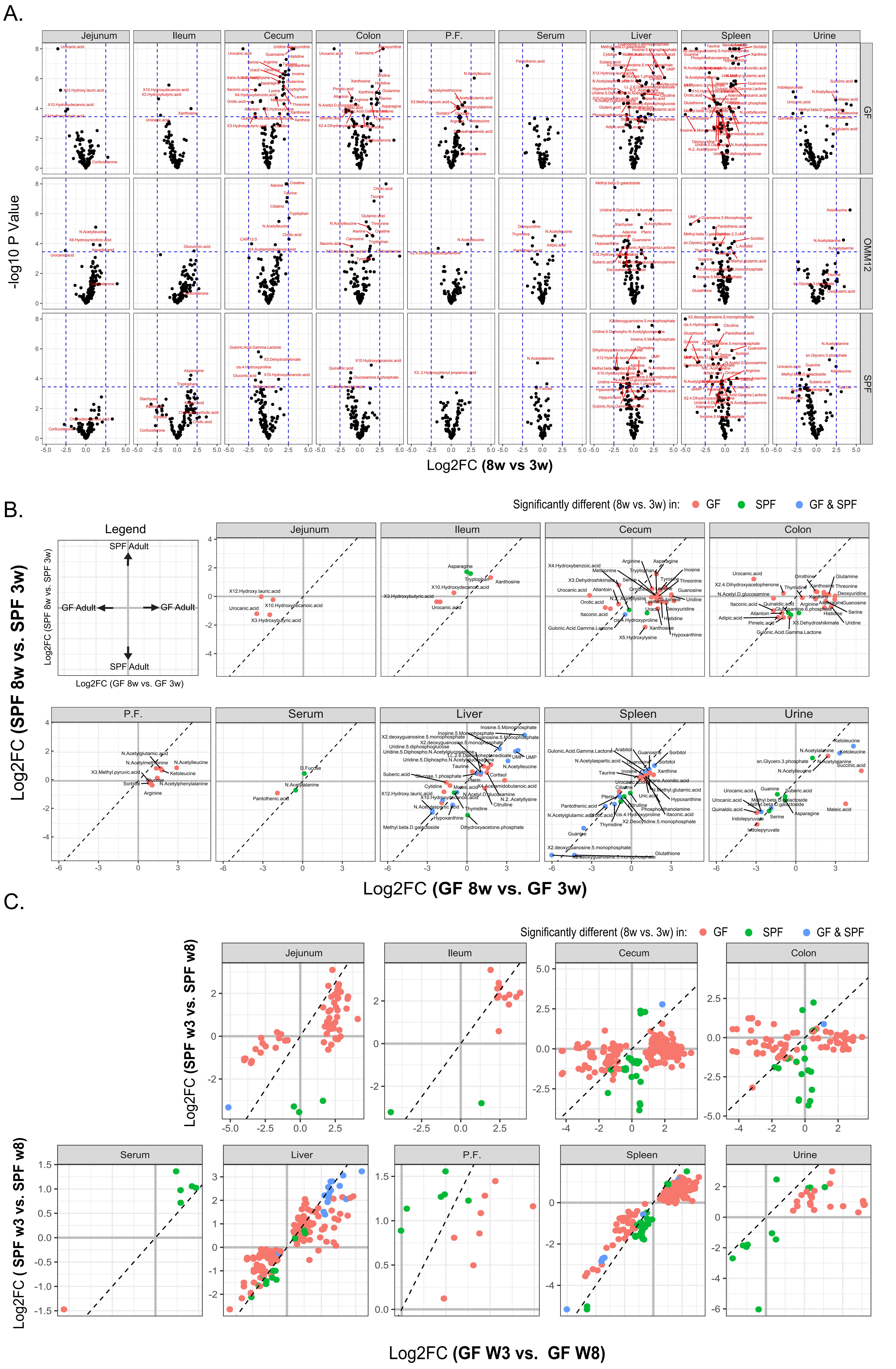

Supplementary Figure 5. Age-specific changes in microbiome-associated metabolism (related to Figure 4). (A) Volcano plot showing differentially abundant metabolites in 8-week vs. 3-week-old mice that were GF or colonized with OMM12 or SPF. Highlighted metabolites were significantly different (PAdj < 0.05) or abs(FC) > 2.5. (B) Labelled biplot showing the Log2FC in metabolites that were significantly different (PAdj < 0.05) in 3-week-old vs. 8-week-old SPF mice or 3-week-old vs. 8-week-old GF mice. Metabolites were significantly different based on age in GF (red) or SPF (green) or both GF and SPF (blue). (C) Biplot of untargeted peaks showing the Log2FC in metabolites that were significantly different (PAdj < 0.05) in 3-week-old vs. 8-week-old SPF mice or 3-week-old vs. 8-week-old GF mice. Metabolites were significantly different based on age in GF (red) or SPF (green) or both GF and SPF (blue). GF=Germ-free, OMM12=Oligo-MM12, SPF=specific pathogen free, P.F.= peritoneal fluid. Source data are provided as a source data file.

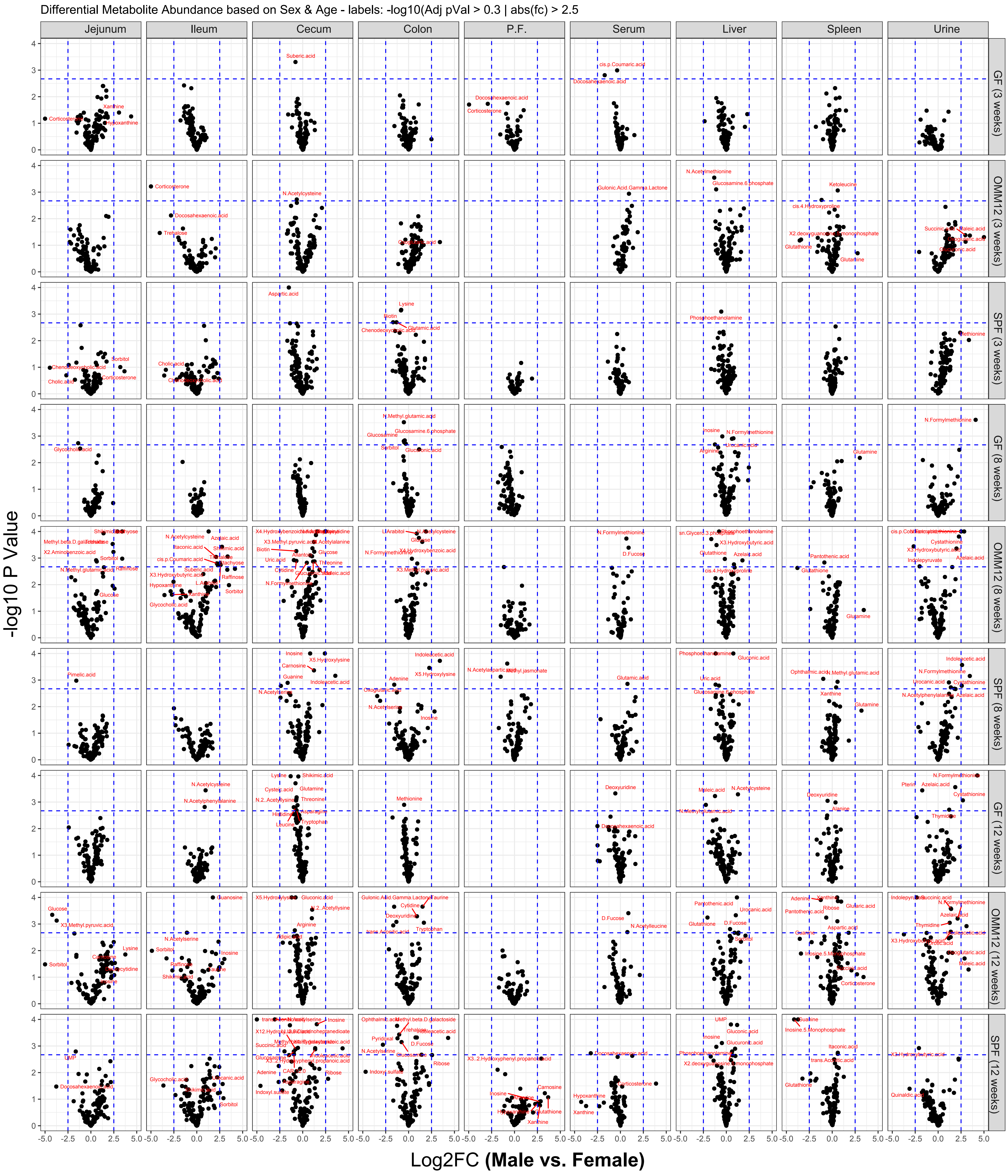

Supplementary Figure 6. Sex-specific changes in microbiota-associated metabolism (related for Figure 5). Volcano plots showing changes in male vs. female mice at 3-, 8-, and 12-weeks in GF, OMM12 and SPF colonized mice. Highlighted metabolites had PAdj < 0.3 or abs(Log2FC) > 2.5. Data represents n=3-4 mice /group. Blank plots are where there were <3 samples in one of the comparison groups. GF=germ-free, OMM12=Oligo-MM12, SPF=specific pathogen free, P.F. = peritoneal fluid. Source data are provided as a source data file.

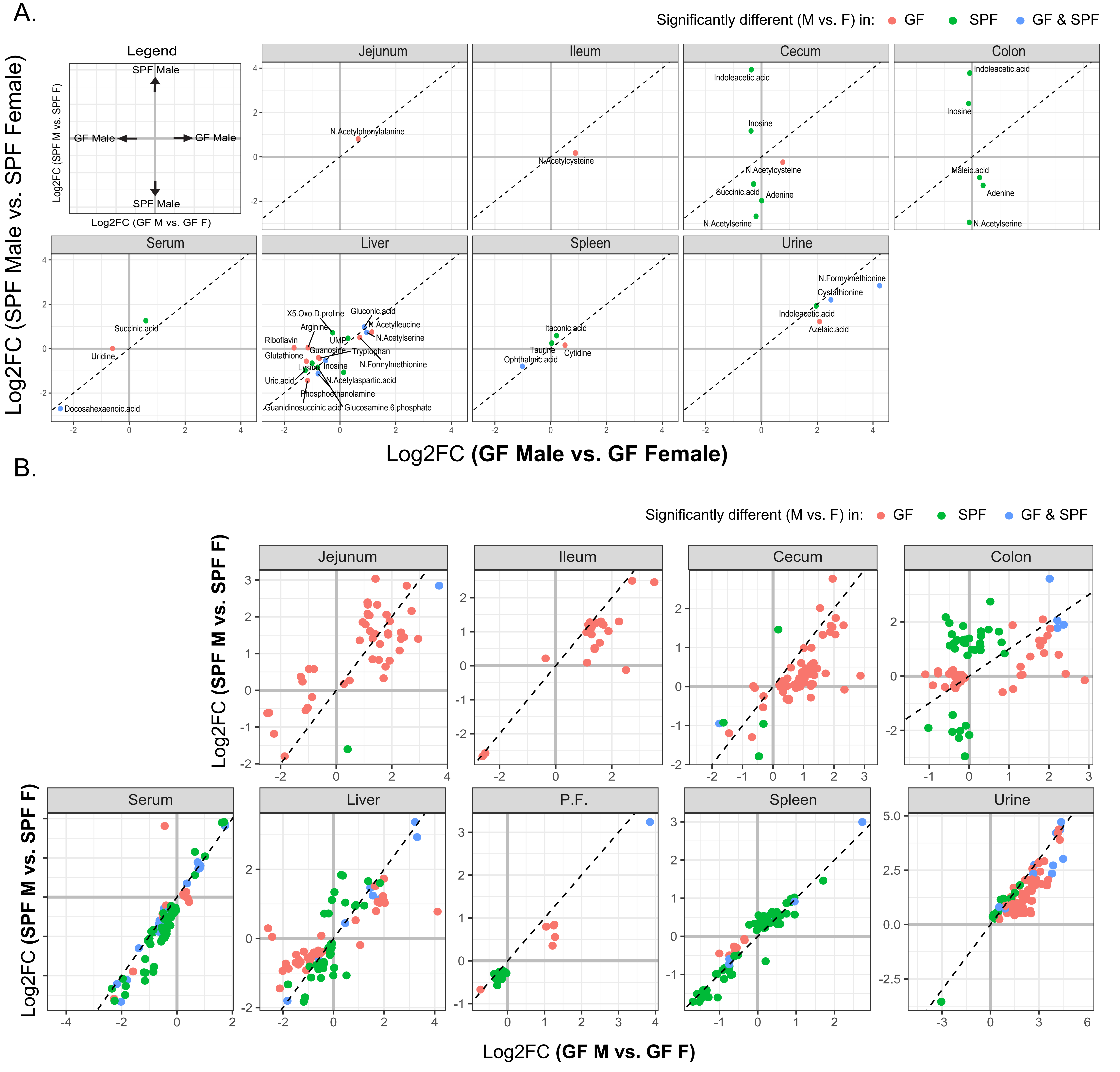

Supplementary Figure 7. Sex-specific changes in microbiota-associated metabolism (related to Figure 5). (A) Labelled biplot showing the Log2FC in metabolites that were significantly different (PAdj < 0.05) in male vs. female SPF mice or male vs. female GF mice. Metabolites were significantly different based on age in either GF (red) or SPF (green) or in both GF and SPF (blue). (B) Biplot from untargeted data showing Log2FC in metabolites that were significantly different (PAdj < 0.05) in male and female SPF mice of male vs. female GF mice. Metabolites were significantly different based on age in either GF (red) or SPF (green) or in both GF and SPF mice (blue). Data represents 7-8 mice / group. GF=germ-free, SPF=specific pathogen free, P.F. = peritoneal fluid, M=male, F=female. Source data are provided as a source data file.

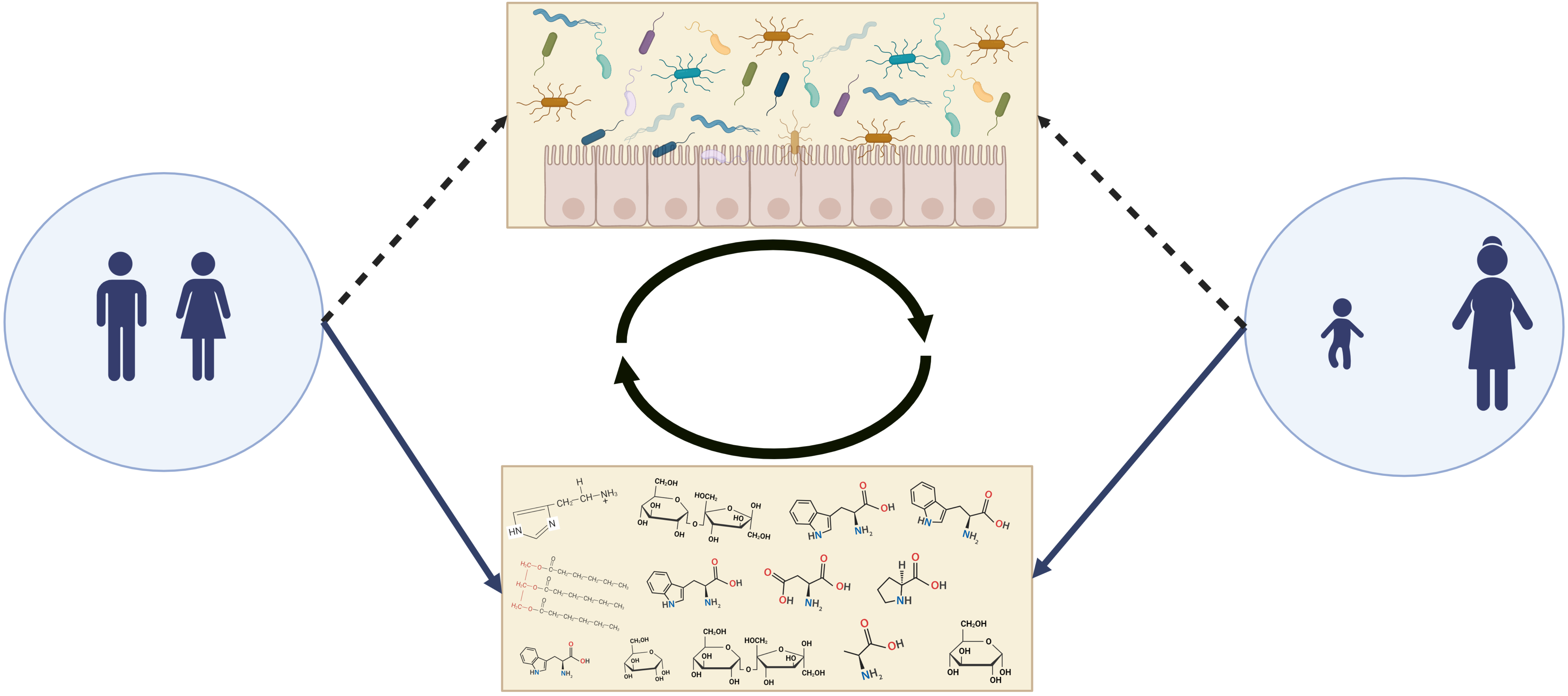

Supplementary Figure 8: Summary Figure. Microbiota alter metabolism in an age- and sex- dependent manner. Composition of the microbiota has a considerable impact on metabolism within the GIT and affects metabolism throughout the organism. Age impacts the metabolome and has a bigger effect than microbiota in several body sites. Biological sex also has a significant effect on metabolism at several body sites. Microbial colonization differentially affects metabolism based on the age and sex of the organism and as such, age and sex should be considered when evaluating microbe-associated metabolic changes. Understanding microbe-host-metabolite interactions sets the stage to harness the power of these connections in order to promote health and improve therapeutics in disease. This figure was generated using biorender.com under license.

Supplementary Table 1. Summary of estimated factor effect size on overall metabolome.
Analysis of the effect of site microbiota, age and sex on metabolome.
Source data are provided as a source data file.

Factor	D.F.	SumOfSqs	R^2	F	Pr(>F)
Site	8	43956.8620	0.5300	92.4031	0.0001
Microbiota	2	2811.5525	0.0339	23.6410	0.0001
Age	2	998.8788	0.0120	8.3991	0.0001
Sex	1	206.2045	0.0025	3.4678	0.0007
Residual	588	34964.5022	0.4216	NA	NA
Total	601	82938.0000	1.0000	NA	NA

Supplementary Table 2. Summary of estimated factor effect size on overall metabolome. Analysis of the effect of microbiota, age and sex on metabolome in individual sites (related to Figure 2). Source data are provided as a source data file.

Site	Factor	P-Value	R2		Site	Factor	P-Value	R2
**Jejunum**	Microbiota	1.00E-04	0.1696		**P.F.**	Microbiota	2.00E-04	0.1353
	Age	1.00E-04	0.0809			Age	1.00E-04	0.1568
	Sex	0.0231	0.026			Sex	0.0298	0.0347
	Residual	NA	0.7235			Residual	NA	0.6732
	Total	NA	1			Total	NA	1
**Ileum**	Microbiota	1.00E-04	0.18		**Serum**	Microbiota	1.00E-04	0.1035
	Age	9.00E-04	0.0697			Age	1.00E-04	0.1556
	Sex	0.0797	0.0204			Sex	1.00E-04	0.0521
	Residual	NA	0.7299			Residual	NA	0.6888
	Total	NA	1			Total	NA	1
**Cecum**	Microbiota	1.00E-04	0.4905		**Liver**	Microbiota	1.00E-04	0.0764
	Age	1.00E-04	0.0688			Age	1.00E-04	0.2566
	Sex	0.0119	0.0196			Sex	1.00E-04	0.0759
	Residual	NA	0.4212			Residual	NA	0.5911
	Total	NA	1			Total	NA	1
**Colon**	Microbiota	1.00E-04	0.442		**Spleen**	Microbiota	1.00E-04	0.0855
	Age	1.00E-04	0.0841			Age	1.00E-04	0.2927
	Sex	0.0218	0.0176			Sex	9.00E-04	0.0422
	Residual	NA	0.4562			Residual	NA	0.5797
	Total	NA	1			Total	NA	1
					**Urine**	Microbiota	1.00E-04	0.1187
						Age	1.00E-04	0.1501
						Sex	0.001	0.0525
						Residual	NA	0.6788
						Total	NA	1
