# Supplementary material for: Microbiota alters the metabolome in an age- and sex- dependent manner in mice
Source: Nat Commun. 2023 Mar 11;14:1348. doi: 10.1038/s41467-023-37055-1 (PMC10008592; doi:10.1038/s41467-023-37055-1)
Supplement: Supplementary file 3 — Reporting Summary [file 41467_2023_37055_MOESM3_ESM.pdf]

nature portfolio

Corresponding author(s): McCoy, Kathy

Last updated by author(s): 2023/02/04

Reporting Summary

Nature Portfolio wishes to improve the reproducibility of the work that we publish. This form provides structure for consistency and transparency in reporting. For further information on Nature Portfolio policies, see our Editorial Policies and the Editorial Policy Checklist.

Statistics

For all statistical analyses, confirm that the following items are present in the figure legend, table legend, main text, or Methods section.

n/a	Confirmed	
☐	☒	The exact sample size (n) for each experimental group/condition, given as a discrete number and unit of measurement
☐	☒	A statement on whether measurements were taken from distinct samples or whether the same sample was measured repeatedly
☐	☒	The statistical test(s) used AND whether they are one- or two-sided<br>*Only common tests should be described solely by name; describe more complex techniques in the Methods section.*
☐	☒	A description of all covariates tested
☐	☒	A description of any assumptions or corrections, such as tests of normality and adjustment for multiple comparisons
☐	☒	A full description of the statistical parameters including central tendency (e.g. means) or other basic estimates (e.g. regression coefficient) AND variation (e.g. standard deviation) or associated estimates of uncertainty (e.g. confidence intervals)
☐	☒	For null hypothesis testing, the test statistic (e.g. F, t, r) with confidence intervals, effect sizes, degrees of freedom and P value noted<br>*Give P values as exact values whenever suitable.*
☒	☐	For Bayesian analysis, information on the choice of priors and Markov chain Monte Carlo settings
☒	☐	For hierarchical and complex designs, identification of the appropriate level for tests and full reporting of outcomes
☒	☐	Estimates of effect sizes (e.g. Cohen's d, Pearson's r), indicating how they were calculated

Our web collection on statistics for biologists contains articles on many of the points above.

Software and code

Policy information about availability of computer code

Data collection	Data processing was performed using MINT (version 0.1.8.3; mint.resistancedb.org) . Source code and the software application can be found at the following link: https://lewisresearchgroup.github.io/ms-mint-app/gui/
Data analysis	Example code for reproducing plots is located at the following repository: (https://github.com/kirbrown/microbiome-metabolites)

For manuscripts utilizing custom algorithms or software that are central to the research but not yet described in published literature, software must be made available to editors and reviewers. We strongly encourage code deposition in a community repository (e.g. GitHub). See the Nature Portfolio guidelines for submitting code & software for further information.

Data

Policy information about availability of data

All manuscripts must include a data availability statement. This statement should provide the following information, where applicable:

- Accession codes, unique identifiers, or web links for publicly available datasets
- A description of any restrictions on data availability
- For clinical datasets or third party data, please ensure that the statement adheres to our policy

Raw and processed data is available at the NIH Common Fund's National Metabolomics Data Repository (NMDR) website, the Metabolomics Workbench, https://www.metabolomicsworkbench.org where it has been assigned Project IDs PR001468 (DOI: http://dx.doi.org/10.21228/M8ZT5R). 16S amplicon sequencing data have been deposited at NCBI SRA: PRJNA931649(https://www.ncbi.nlm.nih.gov/sra/PRJNA931649).

Human research participants

Policy information about studies involving human research participants and Sex and Gender in Research.

Reporting on sex and gender	N/A
Population characteristics	N/A
Recruitment	N/A
Ethics oversight	N/A

Note that full information on the approval of the study protocol must also be provided in the manuscript.

Field-specific reporting

Please select the one below that is the best fit for your research. If you are not sure, read the appropriate sections before making your selection.

☒ Life sciences ☐ Behavioural & social sciences ☐ Ecological, evolutionary & environmental sciences

For a reference copy of the document with all sections, see nature.com/documents/nr-reporting-summary-flat.pdf

Life sciences study design

All studies must disclose on these points even when the disclosure is negative.

Sample size	Sample size was not predetermined based on power analysis. A small pilot experiment showed biologically interesting results and so a larger scale experiment was performed based on results from the pilot experiment.
Data exclusions	Data were excluded if they presented grossly different metabolite profiles and this could be attributed to a technical error.
Replication	All groups contained n=4 mice per group. All replicates were successful with no replicates excluded.
Randomization	Due to limitations with microbiome cross-contamination, mice could not be randomized into cages. However, all mice were age and sex matched and of identical genetic background. All samples were collected processed uniformly.
Blinding	During sample collection and processing and downstream analysis there was no subjective component. For peak integration and assignment, the person that performed this analysis was blinded to samples groups.

Reporting for specific materials, systems and methods

We require information from authors about some types of materials, experimental systems and methods used in many studies. Here, indicate whether each material, system or method listed is relevant to your study. If you are not sure if a list item applies to your research, read the appropriate section before selecting a response.

Materials & experimental systems

n/a	Involved in the study	
☒	☐	Antibodies
☒	☐	Eukaryotic cell lines
☒	☐	Palaeontology and archaeology
☐	☒	Animals and other organisms
☒	☐	Clinical data
☒	☐	Dual use research of concern

Methods

n/a	Involved in the study	
☒	☐	ChIP-seq
☒	☐	Flow cytometry
☒	☐	MRI-based neuroimaging

Animals and other research organisms

Policy information about studies involving animals; ARRIVE guidelines recommended for reporting animal research, and Sex and Gender in Research

Laboratory animals	C57BL6/J mice, aged 3-12 weeks

Wild animals	Wild animals were not used in this study
Reporting on sex	Equal numbers of male and female mice were used.
Field-collected samples	This study did not involve samples collected from the field.
Ethics oversight	All animal experiments were performed in accordance with the guidelines set by the Canadian Council for Animal Care and all protocols (AC17-0090 and AC17-0011) were approved by the University of Calgary Health Science Animal Care Committee.

Note that full information on the approval of the study protocol must also be provided in the manuscript.
